# Influence of electrospinning parameters on biopolymers nanofibers, with emphasis on cellulose & chitosan

**DOI:** 10.1016/j.heliyon.2023.e17051

**Published:** 2023-06-07

**Authors:** Abdallah Refate, Yehia Mohamed, Mariam Mohamed, Maiada Sobhy, Karim Samhy, Omar Khaled, Khaled Eidaroos, Hazem Batikh, Emad El-Kashif, Samah El-Khatib, Sherif Mehanny

**Affiliations:** a Mechanical Design & Production Dept., Faculty of Engineering, Cairo University, Giza, Egypt; b Mechatronics Program, Faculty of Engineering, Cairo University, Giza, Egypt; c Electronics and Communication Dept., Faculty of Engineering, Cairo University, Giza, Egypt; d Mechanical Engineering Dept., Faculty of Engineering & Technology, Future University in Egypt, 11835 Cairo, Egypt

**Keywords:** Nanofibers, Electrospinning, Cellulose, Chitosan, Nanocellulose, Membrane

## Abstract

**Background:**

Electrospinning is an effective method for producing high-quality biopolymer nanofibers, such as cellulose and chitosan. Cellulose nanofibers have excellent mechanical properties and biocompatibility, making them a promising material for tissue engineering. Chitosan nanofibers are biodegradable, biocompatible, and antimicrobial, making them ideal for biomedical applications. The electrospinning parameters, including solution concentration, power supply voltage, orifice diameter, temperature, humidity, and flow rate, play a crucial role in determining the nanofiber diameter, morphology, and mechanical properties, as well as their suitability for various applications.

**Objective:**

This systematic review aims to synthesize and evaluate the current evidence on the influence of electrospinning parameters on the production and properties of cellulose and chitosan nanofibers.

**Methods:**

A comprehensive search of electronic databases was conducted to identify relevant studies. The inclusion criteria were studies that investigated the effect of electrospinning parameters on cellulose and chitosan nanofibers.

**Results:**

It was found that for cellulose, the average fiber diameter increased with increasing each of solution concentration, power supply voltage, orifice diameter, temperature, and humidity. Contrary to tip - collector distance and some optimal points in temperature, where average fiber diameter decreased. For chitosan, the change in voltage and tip to collector distance did not alter the average fiber diameter except for some readings of voltage, which behaved differently. On the other hand, the average fiber diameter increased with increasing flow rate.

**Conclusion:**

The review highlights the importance of considering electrospinning parameters in the production of high-quality biopolymer nanofibers and provides insights into the optimization of these parameters for specific applications. This review also highlights the need for further research to better understand the underlying mechanisms of electrospinning and to optimize the process to produce biopolymer nanofibers with improved properties.

## 1 Introduction

Biopolymers have received much interest in recent years due to their lower environmental impact compared to the use of fossil fuels and other pollutants, which have a direct harmful impact on the environmental system’s stability [[1], [2], [3]]. They’re utilized in a variety of applications, including medicine, food packaging, and cosmetics, where they may substitute any petroleum-based substance. Polynucleotides, polypeptides, and polysaccharides are three different types of biopolymers. Cellulose is a polysaccharide-type biopolymer that is extremely crystalline and extensively abundant biopolymer on the terrestrial/aquatic environments, since it can be found in all plant/algal cells (a part of its cell wall composition) [4]. Because it is so readily available in nature, it has been widely exploited in the field of electrospinning. However, it is limited by its inability to dissolve in common solvents such as water (although some experiments have shown that it can slightly dissolve in water); moreover, it cannot melt, making it processable only in solution form [5,6]. Cellulose may also be modified by introducing hydroxyl groups on the cellulose backbone, and the resulting derivatives (acetate, hydroxyl propyl, and so on) can be utilized to make nanofibers [[7], [51]]. Both cellulose and its derivatives are important raw materials in the electrospinning process, where the resulting fibers made their way into a variety of biomedical applications, igniting the interest in cellulose and its derivatives.

Chitosan is another significant biopolymer that is also of polysaccharide nature. It is biodiverse, biocompatible, and biodegradable [8]. Chitosan is made by treating chitin shells of shrimps and other crustaceans with an alkaline substance such as sodium hydroxide. It is considered the second most abundant biopolymer after cellulose. Chitosan is insoluble in water, alkali, and mineral acidic systems. It is, however, soluble in organic acids. The study and development of these materials at the nanoscale is one of the current fastest developing scientific fields due to its enormous potential for developing novel resulting derivatives with advanced applications [9,10]. Many diverse sciences and engineering disciplines, such as electronics, material science, and polymer engineering, have been significantly influenced by nanotechnology. Nanofibers are used in Nano-catalysis, tissue scaffolds, protective clothing, filtration, and optical electronics, among other things, due to their large surface area and porosity [[11], [12], [52]]. Electrospinning is a viable technique for producing nanofibers. It has gotten a lot of attention in the last years, not only because of its versatility in spinning a wide range of polymeric fibers, high specific surface area, ease of surface functionality, and interfibrous pore sizes [13], but also because of its consistency in creating fibers using polymer melts or solution of both natural and synthetic polymers [[14], [15], [16]]. The electrospinning technique produces electrically charged jets from a polymer solution or melts using a high-voltage electric field. The evaporation of the solvent causes the solution or melt to dry, resulting in nanofibers [13]. A positively charged collector, which might be a flat surface or a revolving drum, attracts the highly charged fibers [9,17]. Fiber is subjected to a collection of tensile, gravitational, aerodynamic, rheological, and inertial forces in traditional spinning procedures. Tensile force induce**d** by Electric field created axial direction of polymeric flow, yielding spinning operation [9].

The aim of this systematic review was, therefore, to study the influence of electrospinning parameters on biopolymers nanofibers, with emphasis on cellulose & chitosan.

a) What’s the effect of material parameters on the output chitosan and cellulose nanofibers resulting from the electrospinning process?
b) What’s the effect of machine parameters on the output chitosan and cellulose nanofibers resulting from the electrospinning process?
c) What’s the effect of ambient parameters on the output chitosan and cellulose nanofibers resulting from the electrospinning process?

## 2 Methods

### 2.1 Review protocol

Preferred Reporting Items for Systematic Reviews and Meta-Analyses (PRISMA) were used to identify eligibility and selecting of articles for this review.

### 2.2 Search strategy

The literature search started from September 1st, 2022, to February 5th, 2023, with the use of electronic databases like Google Scholar, Science Direct, Web of Sciences, Scopus, PubMed, and official websites of different organizations and universities. Published articles written in English language were considered without restriction to year of publication. Electrospinning, Biopolymers Nanofibers, Cellulose & Chitosan, Electrospinning Parameters, Cellulose Nanofibers, Chitosan Nanofibers were the search terms used. These terms were used in an advanced PubMed search to widen the search that included all fields [All fields] in record. Furthermore, Boolean operators (AND, OR) were appropriately employed for identifying research. For all the papers included in this review, the searching words that is used for advanced search was as: “Electrospinning” OR “Biopolymers Nanofibers” [All fields] OR “Nanofibers” [All fields] OR “Electrospinning Parameters” [All fields] AND “Cellulose & Chitosan” [All fields].

### 2.3 Eligibility criteria

#### 2.3.1 Inclusion criteria

All prospective and retrospective observational studies (cross-sectional, case controls, and cohort) articles conducted in any field of Cellulose & Chitosan and written only in English language were included if they reported the influence of electrospinning parameters on biopolymers nanofibers.

#### 2.3.2 Exclusion criteria

Those studies did not report our variables of interest or comprised incomplete information (parameters, method of preparation, and findings). Articles where the full text cannot be accessed were excluded from this systematic review.

#### 2.3.3 Evaluation of articles quality

The quality of each article was evaluated by using a 14-points checklist. ‘High quality’ for an article with score > and = 70%. A score of 69 to 51% considered as “moderate quality”, less than or equal to 50% were considered “Poor quality”. Two authors scored each article individually and the mean value of the results was used. No article got excluded due to inferior quality, as all articles scored more than 50%.

### 2.4 Data extraction and analysis

Using Microsoft Excel 2019, authors created a data extraction tool to collect all the needed data from selected articles. Data related to characteristics of the articles such Parameter, Material, Methods and Preparations, Results, Findings were extracted. Solution concentration and applied volt were also extracted. The data extraction was carried out by two authors independently. When disagreement was encountered by the two authors, a third author was delegated to extract the data. Microsoft Excel 2019 was used to analyze the Effect of the parameters and the results were depicted in charts and tables.

### 2.5 Risk of bias in individual studies

This study design was made to determine the risk of bias of individual. Validity of the data were assured for each outcome by identifying the methods and analyses that were employed to assess the outcomes. Both study and outcome level information were used in synthesis.

### 2.6 Summary measures and synthesis of results

As the effectiveness was presented in percentage without always mentioning the means and confidence intervals, the principal summary measures could not be estimated.

### 2.7 Risk of bias across studies

After assessing the individual study, the bias across the studies was evaluated. The risks of bias included studies from publication. Selection and reporting are described in the discussion section of the review.

## 3 Results

### 3.1 Characteristics of the studies included

**Fig. 1:**
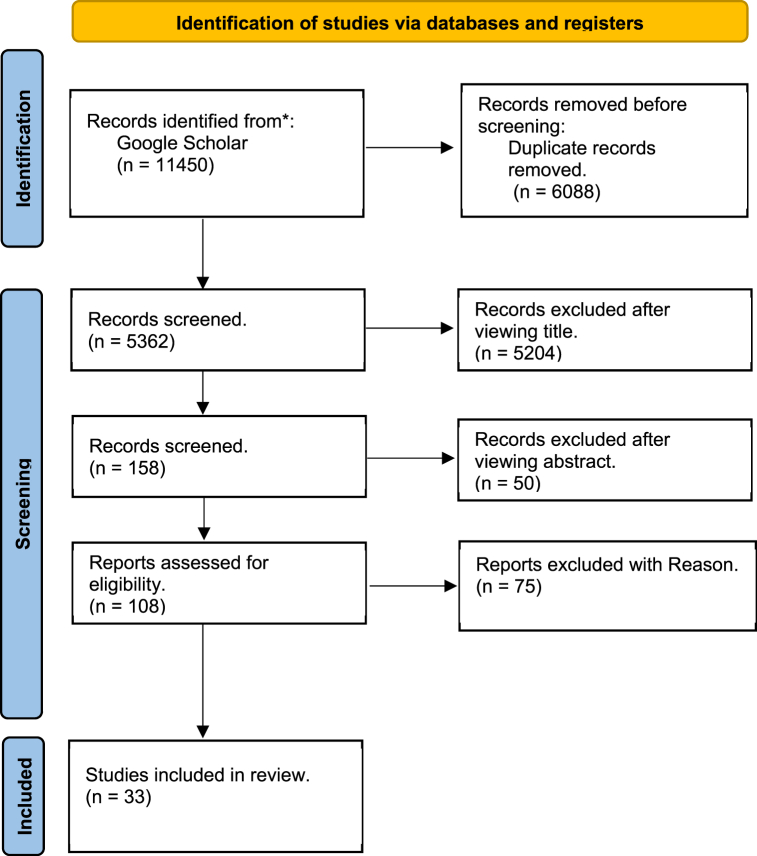
Flow diagram of articles searching, screening and selection process for our systematic review.

During our advanced research, we identified 11,450 articles, and we used the PRISMA flow diagram method (Fig. 1) to demonstrate our search procedure. After removing duplicates, we screened 5362 articles for eligibility. Finally, we found 33 studies that met the eligibility criteria and were included in this review.

### 3.2 Principles of electrospinning method

The electrospinning technique was initially developed by Rayleigh in 1897 and presented in detail by Zeleny in early 1900 [18]. It is now seen as a critical scientific and commercial enterprise with worldwide economic advantages [19]. Electrospinning has grown exponentially and now became the preferred technique among others to produce nanofibers, because of its simplicity in usage, & cost effectiveness [18]. The electrospinning process starts when electric charges passes through the metallic needle and into the polymer solution. As a result of the charges induction on the polymer droplet, instability is produced within the polymer droplet. At the same time, reciprocal charge repulsion creates a force that resists surface tension (only when the polymer solution has sufficient cohesive force a stable charge jet can be produced) [19]. The spherical droplets deform and take on a conical shape (Taylor cone). As the electric field increases, internal and external charge forces cause the liquid jet to whip in the direction of the collector during the operation [21]. This whipping motion causes the polymer chains in the solution to stretch and glide past each other, resulting in ultrafine nanofibers [19,22,23]. They are collected on the metallic collector, which is held at an optimal distance. The most frequent way to collect electrospinning nanofibers is in the form of randomly oriented or parallel-aligned mats. When a basic static collecting surface is utilized, randomly oriented fiber mats emerge. While the parallel-aligned fiber mats are collected with a spinning mandrel [19,24]. Nanofibers can also be collected in a linear orientation over an air gap between two parallel plates, fibers align perpendicular to the plates and extend across them when an electric field is created between the two parallel plates. It extends the scope of electrospinning’s practical applications because this technique can gather individual nanofibers [24].

### 3.3 Effects of parameters on electrospinning

The electrospinning process is determined by several parameters. They are divided into three categories: machine parameters, material parameters, and ambient parameters. Applied electric field, distance between needle and collector, flow rate, and needle diameter are machine parameters. Solvent type, polymer concentration, viscosity, and material conductivity are material parameters. Relative humidity and temperature are ambient parameters. All these parameters have an impact on the production of smooth, bead-free electrospinning fibers, therefore, it is important to fully understand the impacts of all of these key parameters to obtain a better knowledge of the electrospinning process and production of polymeric nanofibers [19,25].

## 4 Discussion

### 4.1 Material parameters (effects of polymer concentration and solution viscosity)

**Fig. 2:**
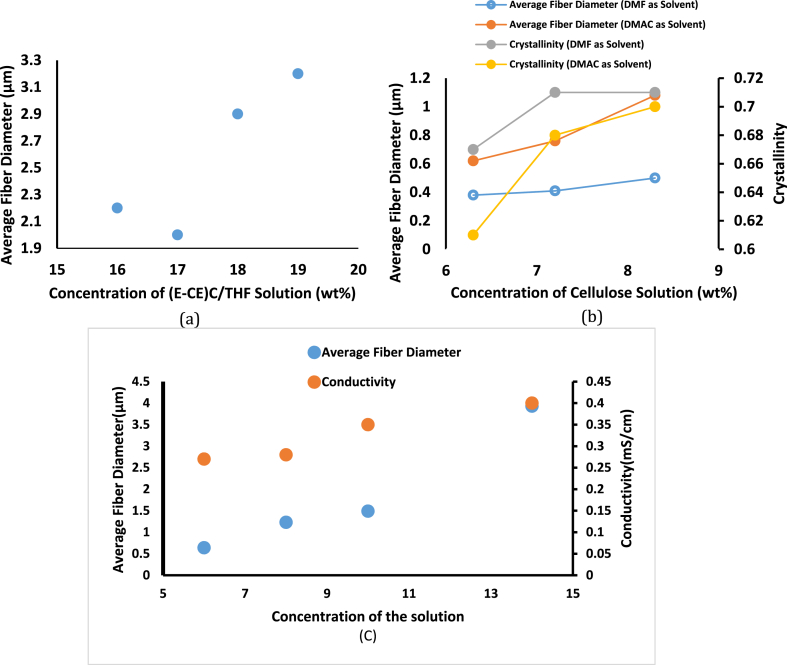
Effect of solution concentration for (a) (E-CE)C/THF solution on Average Fiber Diameter, for (b) Cellulose solution on Average Fiber Diameter and Crystallinity and (c) chitosan/chloroform solution on Average Fiber Diameter and Conductivity [26,27].

The electrospinning method is based on the phenomenon of a charged jet extending uniaxially. Changing the concentration of the polymeric solution has a tremendous impact on the stretching of the charged jet. For example, when the concentration of the polymeric solution is low, the applied electric field and surface tension cause the entangled polymer chains to break up before reaching the collector. Beads or beaded nanofibers are formed because of these fragments; therefore, at very low concentrations, the average fiber diameter is high until the critical value of concentration, as explained in Table 1 [22,27,49]. On the other hand, the viscosity of the polymeric solution increases as the concentration of the solution increases, and the number of macromolecular chains in the solution increases, as does the macromolecular chain entanglement. The surface tension of the solution levels up with increasing concentration. Because the repulsion among the charges in the jet must be higher than the surface tension when the jet splits, increasing the surface tension can also prevent the charged solution jet from splitting. Therefore, as the concentration of the solution increases, the average diameter of the fibers boosts up considerably, and the diameter distribution widens, see Fig. 2(a–c) [26,27]. Moreover, increasing the concentration above a critical value (the concentration at which beadless homogeneous nanofibers are formed) obstructs the flow of the solution through the needle tip (the polymer solution dries at the tip of the metallic needle and blocks it), resulting in defective or beaded nanofibers [19,22,49] It is also noted that there is a difference in values when using different solvents, as explained in Table 1. There are considerable differences in the average and standard deviation of fiber diameter depending on the co-solvent types. In comparison to the fiber web prepared with DMAc, the average and standard deviation of fiber diameter in the fiber web prepared with DMF were significantly lower, as shown in Table 1 [26]. The difference in charge-induced partial polarity between the two co-solvents might explain the effect of the co-solvent type. DMF has a higher partial polarity by electrical charge for spinning than DMAc. During the whipping process, the solution containing DMF would have a higher probability of being properly stretched. Better whipping resulted in finer, more uniform fibers. Crystallinity was higher in the fiber produced with higher co-solvent content [26]. The co-solvent type impacted the degree of crystallinity. The DMF electrospinning fiber has higher crystallinity than the DMAc electrospinning fiber, see Fig. 2(b) [26]. The diffusion rate of the co-solvent type is responsible for this outcome.

**Table 1:** Effect of material parameters (concentration, viscosity, Type of Solvent, surface tension, and Conductivity) on the average fiber diameter.

Material parameters					
Parameter	Material	Methods and Preparations	Results	Findings	Citation
	**Cellulose**	● Dissolving Cellulose (0.628 g) in [C_2_min] [OAc], IL (5 g). ● Adding different amounts of DMF or DMAc (5, 3.75, 2.5 g) with concentrations of (6.3, 7.2, 8.3%) (w%) Respectively. ● Rotating wired cylinder collector. ● Applied voltage 30 kV. ● TCD 150 mm. ● Immersing the spun fiber in Ethanol at 4 °C, 2 h. Drying fiber at 50 °C, 1 day.	● Average fiber diameter for different concentration of the co-solvent is about: (500, 410 and 380 nm) for DMF & (1080, 760 and 620 nm) for DMAc, respectively. ● Degree of crystallinity for different concentration is (0.67, 0.71 and 0.71) for DMF and (0.61, 0.68 and 0.7) for DMAc, respectively.	● The addition of the co-solvent improves the spinnability and led to more uniform and finer fibers. ● Increasing concentration causes increasing in Degree of crystallinity.	[26]
**Concentration, Viscosity and Type of Solvent**	**(E-CE)C**	● (E-CE)C with M_n_ of 9.7 × 10^4^ g/mol was prepared by a reaction of EC and Acrylonitrile with a DS of 2.1 for Ethyl and 0.37 for Cyanoethyl. ● THF was used as the solvent. ● (E-CE)C/THF solution concentrations of (16, 17, 18 and 19) wt% were applied. ● Applied voltage 30 kV. ● The diameter of orifice was 1.2 mm. ● TCD was 150 mm.	● Fiber will be formed only between the concentration of [15–22%], outside this range no formation for the fiber. ● the average fiber diameter was (2200, 2000, 2900 and 3200) nm, with (16, 17, 18 and 19) wt% (E-CE)C/THF solution concentrations respectively.	● Average fiber diameter increased, and the diameter dispersion was broadened with increases in the solution concentration	[27]
	**(CMC) & (PEO)**	● (PEO) with a Mw of 400000 g/mol is used in a mixture with (CMC). ● Different types of CMC were used with different M_W_ and DS as follow: CMC Cekol 30 (A), CMC Cekol 700 (B). ● CMC Cekol 2000S (C), CMC Cekol 500T (D) with Mw of 120, 280, 350, 250 (g/mol) and with DS 0.72, 0.77, 1.24, 0.72 respectively ● PEO and CMC are mixed at a ratio of 1:1 then they are dissolved in water. ● TCD was set to 200 mm. ● Constant voltage at 35 kV.	● At a concentration of 8% for all CMC derivatives, the fibers are straight with an even diameter, the mean diameter of the individual fibers lies between 200 and 250 nm. ● CMC (D) couldn’t be electro-spun at 8% conc. due to its high viscosity, so a lower conc. than 6% was used. ● CMC (A) & (B) lead to the formation of homogenous fibers. ● CMC (C) required a slightly higher voltage (40 kV) due to its high viscosity. ● Nonwoven sheets and individual nanofibers were formed.	● The electrospinning process is directly dependent on the viscosity of the liquid to be operated; high viscosities were found to be in- spinnable unless a change was made on it either concentration wise or voltage wise.	[28]
	**Chitosan & PVA**	● Chitosan 10 of Mv = 2.1 × 10^5^; degree of deacetylation, 0.78 and PVA (Degree of polymerization, approximately 2000; Mn = 8.8 × 10^4^) are used. ● The solvent is a mixture of FA and DW. ● A solution of PVA-DW (9 wt %) was mixed with a chitiosan10-FA solution (7 wt%) with volume ratios 90:10, 70:30, 50:50, and 30:70, respectively. ● Experiment was performed at room temperature. ● A 3 mL syringe with a capillary tip having an inner diameter of 0.6 mm. ● Applied voltage was 15 kV. ● TCD was 150 mm.	● At a ratio of 100:0 chitosan to PVA, no jet had been obtained. ● At a ratio of 90:10 beads started to appear on the collector. ● At a ratio of 70:30 the size of the beads becomes smaller and thin fibers started to appear among these beads. ● For a ratio of 50:50 homogeneous fibers with 120 nm average diameter started to appear. ● For a ratio of 30:70, the fiber was thicker (with an average diameter of 170 nm). ● At 0:100 chitosan to PVA, Average fiber diameter was 470 nm.	● Homogeneity of the produced fibers increases with the decrease of the chitosan percentage in the solution.	[29]
	**Chitosan**	● Chitosan with ≥75% degree of d-acetylation, was used. ● The solvent used was TFA/DCM with different volume ratios. ● The solution was kept under massive and constant magnetic stirring until all the chitosan was dissolved. ● The chitosan concentration was 7% (w/v). ● Diameter of the orifice was 0.5 mm. ● Applied voltage was 25 kV. ● TCD was 150 mm. ● feed rate of 2 mL/h.	● For 60:40, 70:30, 80:20 vol ratio of TFA:DCM the average fiber diameter was 360, 410, 490 nm.	● The increase in the TFA percentage increases the viscosity and conductivity of the solution. ● Lower TFA percentage destroy the homogeneity of the mats.	[30]
	**Chitosan & PVA**	● 4% (w/v) Chitosan flakes were dissolved in 2% (w/v) acetic acid. ● PVA powder was dissolved in deionized water at 90 °C for 2 h. ● The two solutions were mixed. ● Voltage was 15 kV. ● TCD was 150 mm. ● Feed rate was 0.03 mL/h. ● Temperature was 20 °C ● Humidity was 51%.	● For different PVA: Chitosan ratios (100:0, 95:5, 90:10, 80:20, 75:25, 70:30, 50:50) (w:w) the average fiber diameter was 1059, 823.6, 799.4, 637.6, 393.6, 286.2, 119.8 nm. ● When the Chitosan content was more than 50%, the electrospinning process couldn’t occur.	● The increase of the chitosan content leads to the increase of the charge density, which hardens the process of fiber formation. ● The smallest fibers can be obtained with the increasing of chitosan content.	[31]
	**Chitosan**	● Three samples of Chitosan were used with the following M_w_ (30,000, 106,000, 398,000 g/mol) with degree of deacetylation of (56%, 54%, 65%) respectively ● The solution was inserted into a syringe with an orifice diameter of 0.58 mm. ● The flow rate was adjusted to be 1.2 mL/h. ● voltage up to 40 kV.	● At acetic acid concentration of 30% (wt%) Average diameter was found to be 40 nm with large beads. But, at concentration of 90% (wt%) the fiber diameter increased to 130 nm without beads. ● As the concentration of acetic acid increased from 10 to 90% (wt%), Surface Tension decreased from 54.6 to 31.5 dyn/cm	● The known difficulty in electrospinning of Chitosan can be solved by dissolving Chitosan in concentrate acetic acid in water, resulting in low surface tension. ● Acetic acid concentration was the most important parameter as it decreased surface tension and increased charge density without significant effect on viscosity.	[32]
	**CA**	● Cellulose acetate with 39.8% acetyl content and M_w_ of 30,000. ● Acetic acid and water were mixed to make the solvent. ● Cellulose Acetate of 17 wt% was dissolved in acetic acid solution of concentration higher than 70 wt%. ● The applied voltage was 25 kV. ● TCD was adjusted to 100 mm. ● The flow rate was set to 3 mL/h. ● The orifice diameter was 0.84 mm.	● The viscosity of CA solutions increased by the increase of acetic acid up to 80 wt%, and then it decreased. ● The conductivity decreased by the increase of the water content. ● The average fiber diameter was as follow (200, 250, 300, 500, 1300 nm) for acetic acid concentration of (70, 75, 80, 90, 95 wt %)	● The average diameters of the CA nanofibers increases by increasing concentrations of the mixed solvent. ● The viscosity of CA solutions does not affect the average diameter of the nanofibers.	[33]
	**Cellulose**	● Raw cellulose fiber was used. ● The cellulose fiber was dried at 105 °C for 24 h ● Cellulose was dissolved in IL of BMIMAc ≥95% in concentration, (300 mg of cellulose was added to 29.7 g IL). ● Stirred for 72 h at 90 °C and 250 rpm ● The solution was loaded in 10 mL syringe of 0.337 mm needle inner diameter. ● TCD was adjusted to be 25 mm. ● The applied voltage varied from 6 to 12 kV ● Flow rate was 1.36 and 2.38 mL/h ● Temperature inside the syringe was (110 ± 10) ● The process was done at constant relative humidity (65 ± 3%) and temperature of (21 ± 1 °C).	● Stable fiber formed at 2 and 3 wt% concentration ● In case of 1 wt% concentration the Taylor cone was interrupted with the existence of solution droplets. ● In case of 4 wt% concentration the viscosity of the solution was too high which prevent the formation of fibers.	● The viscosity of the tested solution affects its spinnability and fiber formation, intermediate viscosity solution allows stable fiber formation.	[34]
	**CA & CB**	● 12% (w/v) Cellulose acetate of 29–46% acetyl content was dissolved in DMAA with ratio of 4:1 (v/v). ● The CA/DMAA solution was mixed with PEG for 1:1 (w/w) ● CB of (0.7, 1.5 and 2.2 wt%) was mixed with the CA solution ● 20 mL syringe was used ● The applied voltage was 26 kV and −10 Kv. ● The process was done at 24 °C and relative humidity lower than 20%. ● TCD was 10 cm ● Flow rate was 10 mL/h ● The aluminum collector was on cylindrical shape.	● Cylindrical bead free smooth fibers were formed with the following characteristics: - The average fiber diameter was 495, 628, 727, and 831 nm for 0.7, 1.5 and 2.2 wt% of CB respectively. - A few CB small particles were adhered to the resulted fibers. - Pores formed on the surface of the resulted fibers.	● The increase in CB concentration increases the average fiber diameter. ● The pores formed on the surface of the fibers is due to the removal of PEG.	[35]
	**Chitosan**	● Two solutions were used in the experiment chitosan – TFA and chitosan – TFA/DCM ● The first solution was made by mixing 1.12 g of chitosan with 14.9 g of TFA and the final solution concentration was 7 wt%. ● The second solution was made by mixing 1.09 g of chitosan with 10.43 g of TFA dissolved in 3.99 g of DCM at a ratio of 70:30 (TFA: DCM) (v/v), the final solution concentration was 7 wt% ● The two solutions were prepared at room temperature ● The two solutions were left for a night to make a homogeneous solution ● 20 mL syringe was used ● Flow rate was 0.08 mL/h and 0.1 mL/h for the two solutions respectively ● 25 kV was applied ● TCD was 12 cm and 14 cm for the two solutions respectively ● Aluminum collector was used	● Electrospinning of the first solution yields fiber with an average diameter of 95.58 ± 39.28 nm and beads formation was observed in it. ● Electrospinning of the second solution yields bead-free fiber with an average diameter of 907.94 ± 290.18 nm	● The solvent used in the electrospinning process affect the average fiber diameter and the bead formation.	[36]
	**(PLA/CMC/GO-f-COOH)**	● 10 mL of the polymer matrix solution PLA, CMC/GO-COOH, PCGC a syringe with an orifice stainless-steel needle with a diameter of 0.6 mm ● Voltage was 20 kV, ● Flow rate of 0.25 mL/h. ● The distance between the needle and the collector was 20 cm,	● All the nanofibrous membranes exhibited uniform fiber diameter distribution sizes of 350–550 nm. ● The PLA nanofiber had a larger diameter than the PCGC and PCGC@Ag nanofibrous materials	● The increase of thermal treatment result in an increasing in the surface area of the planar surface ● High difference in the morphologies of nanofibrous membranes could clearly be seen, ● The fiber diameters were different for all the nanofibrous materials. ● The nanofibrous membrane had a good distributed pore size, and the PLA sample was densely fibrous more than the other samples.	[37]
	**Chitosan & PEO**	● Chitosan (5% w/v) and PEO (2.5% w/v) solutions in 70% v/v acetic acid were mixed to obtain different Chitosan:PEO respective weight ratios of 9:1, 8:2, 7:3, 6:4, and 5:5. ● A pure Chitosan solution (5% w/v) was tried to obtain electrospinning nanofibers. ● 5 mL syringe with a needle (gauge 20). ● The flow rate was 0.3 mL/h ● The distance between the collector and the needle tip was 15 cm. ● Voltage range of 25–28 kV	● Nanofibers based on 8:2 Chitosan:PEO exhibited the smallest diameter (119.17 ± 22.05 nm) and the greatest mucoadhesion (22.82 ± 3.21 g/cm2) ● A spinnable solution containing Chitosan and PEO at a respective weight ratio of 9:1 produced nanofibers with an average diameter of 135.54 ± 67.48 nm	● The high viscosity of the pure Chitosan solution is owing to the strong hydrogen bonding between its OH and NH2 groups. ● PEO molecules linked to chitosan backbone could disrupt chitosan chain self-association and reduce chitosan solution viscosity ● Small and flexible PEO chains can also lie down along the rigid chitosan macromolecules smoothing their flow.	[38]
	**Chitosan & PEO**	● Low molecular weight chitosan with a viscosity average molecular mass ${\bar{M}}_{v}$ = 540 kDa and a degree of deacetylation DD = 78% ● Chitosan powder was dissolved using a 5% v/v acetic acid water solution to obtain a polymer concentration of 2.5%, 3.0% and 3.5% wt ● PEO powder was added in a Chitosan/PEO mass ratio of 50/50 to obtain a final polymer concentration of 5%, 6% and 7 % wt. ● A solution flow rate of 0.15 mL/h ● A voltage of 17.5 kV ● A needle-flat collector distance of 20 cm.	● The sample obtained by Chitosan:PEO of 50:50, 5 % wt solution with a diameter ranging from 500 nm up to 2 μm and connecting the nanofibers, by an average dimension of 150 ± 30 nm. ● The other two samples are both characterized by a similar structure with homogenous and smooth nanofibers of around 300 ± 30 nm ● However, the mat obtained by Chitosan:PEO of 50:50 6 % wt formulation presents a high number of defects (e.g. beads, collapsed structures, low fibre density regions), whereas the one obtained by Chitosan:PEO of 50:50, 7 % wt. formulation shows the best structure in terms of fiber homogeneity.	● The spinnability increased with solution viscosity, as well as the improvement of homogeneity and overall morphology of nanofibers. ● Decrease of the viscosity as the shear rate increases due to the progressive orientation and disentangle of the chains ● The increase of viscosity of the spinning solution often leads to the increase of polymer concentration, so that the fiber diameter increases and the bead like structure disappear.	[39]
**Surface Tension**	**(E-CE)C**	● (E-CE)C with M_n_ weight of 9.7 × 10^4^ g/mol, was prepared by a reaction of EC and Acrylonitrile with a DS of 2.1 for Ethyl and 0.37 for Cyanoethyl. ● THF was used as the solvent. ● The applied voltage is 30 kV. ● The diameter of orifice was 1.2 mm. ● TCD was adjusted to be 150 mm.	● When the concentration of the (E-CE)C/THF solutions was lower than 15 wt%, the fibers could not be formed by electrospinning because of the low surface tension and viscosity. ● At solution concentrations greater than 22 wt%, the solution jet could not erupt because of the high surface tension and viscosity.	● Surface tension is observed to be increased by increasing the respective concentration. ● By increasing surface tension, average diameter of the fibers increases and the diameter distribution becomes wider with increasing the concentration.	[27]
	**H-Chitosan**	● H-chitosan was prepared by a heterogeneous acylation reaction between Chitosan and hexanoyl Chloride. ● H-chitosan in chloroform to produce a solution of concentration between (4–14%) (w/v) ● Voltage ranged from 12 kV. ● Needle was tilted at 45°. ● TCD was 120 mm.	● When the conc. of H-chitosan was 4% (w/v), the conductivity was 0.25 mS/cm only beads formed at this concentration. ● At 6% conc. The conductivity was 0.27 mS/cm, and the average fiber diameter was 640 ± 360 nm. ● At 8%, the conductivity was 0.28 mS/cm, and the average fiber diameter was 1230 ± 670 nm. ● At 10%, conductivity was 0.35 mS/cm, and the average fiber dimeter was 1490 ± 690 nm. ● At 14%, the conductivity was 0.4 mS/cm, and the average fiber diameter was 3930 ± 1820 nm.	● Conductivity of the solution increases with the increase of the H-chitosan concentration, which happens as a reason of increasing the hydroxyl group in the solution. ● The average diameter increased with the increasing of the conductivity of the solution	[40]
	**Conductivity** **Chitosan & PEO**	● Chitosan and PEO solutions with 2–8% concentration was dissolved in acetic acid aqueous of 2 wt%. ● Mass ratios of Chitosan:PEO were 1:1, 2:1 and 5:1. ● Chitosan solutions with concentrations 1 wt% to 6 wt% and PEO solutions with 1 wt% to 3 wt% in aqueous 2 wt% acetic acid ● stirred overnight at room temperature. ● a 50 mL syringe with a metal capillary. ● The applied voltage was set to be 15 kV. ● The flowrate was adjusted to 0.1 mL/h. ● TCD was adjusted to be 200 mm.	● Conductivity of Chitosan was found to be 2.6–9.6 mS/cm, and conductivity of PEO was found to be 0.85–1.22 S/cm. ● ● With the increase of Chitosan concentration, Conductivity of Chitosan solutions significantly increased. The viscosity also increased at concentrations of 1–6 wt%. ● With the increase of PEO concentration, the conductivity of Chitosan/PEO solutions decreased. ● With the increase of PEO concentrations, Surface Tension of PEO solutions reduced. Fibers were produced with an average diameter of 124 nm at 6 wt% solutions (1:1) (w/w).	● Ultrafine fibers could only be electrospinning from chitosan solution in aqueous acetic acid when PEO was added. ● Conductivity increases with the increase of Chitosan concentration.	[41]

### 4.2 Machine parameters

#### 4.2.1 Effect of applied voltage

**Fig. 3:**
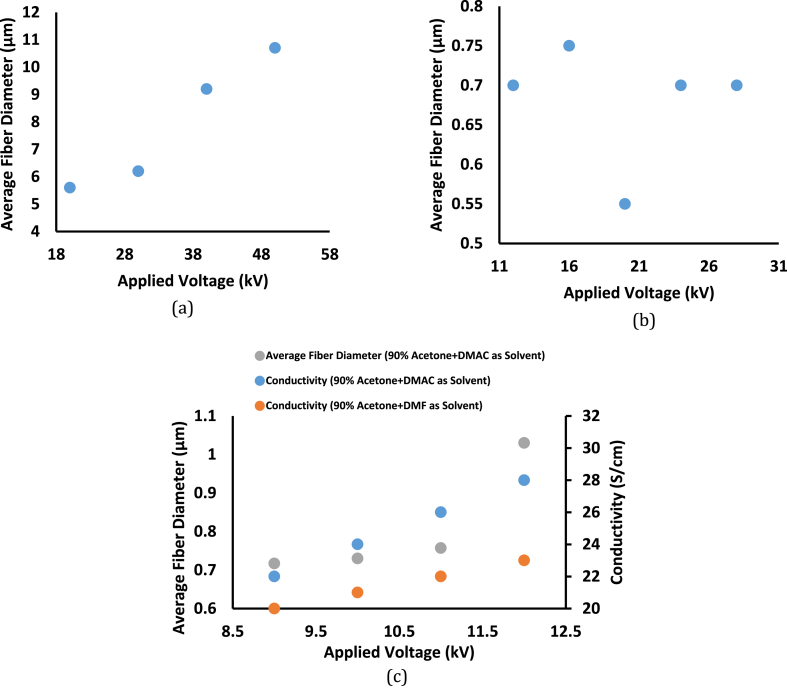
Effect of Applied Voltage (a) for 17 wt% of (E-CE)C/THF Solution on Average Fiber Diameter, (b) for 8% of Chitosan Collagen dissolved in HFIP/TFA on Average Fiber Diameter and (c) for (Cellulose Acetate:DMAC or DMF) (1:9 wt/wt) on Conductivity and Average Fiber Diameter [27,42].

Fiber diameter increases with the increase of the applied voltage, as depicted in Fig. 3(a–c) [27,42]. The decrease in the size of the Taylor cone and rise in the jet velocity for the same flow rate is related to the increased diameter and formation of beads or beaded nanofibers with an increase in the applied voltage [19,25,50]. With decreasing electrostatic field voltage, the average diameter of the fiber decreases, and the dispersion of diameters narrows. The influence of the electrostatic field on the charged solution jet decreases as the voltage of the field decreases, and the jet’s flight speed decreases, consequently the time it takes to go from the anode to the collector increases. The charged jet’s ability to divide and elongate increases, making it easier to create thin fibers with a narrow diameter distribution [27,42,50]. While for chitosan, voltage doesn’t have a significant effect on the fiber diameter, see Fig. 3(b) [43]. Some parameters, such as the mass of polymer fed out from the needle’s tip, the elongation level of a jet caused by an electrical force, and the shape of a jet may be affected by the applied voltage (a single or multiple jets). The resulting diameter of electrospinning fibers may be controlled by a balance of several parameters, as explained in Table 2 [43]. For any type of solvent, conductivity increases by increasing applied voltage, due to the increase of electrostatic forces on the jet (Fig. 3(c)) [42].

**Table 2:** Effect of machine parameters (applied voltage, flow rate, TCD, and Orifice Diameter) on the average fiber diameter.

Machine parameters					
Parameter	Material	Methods and Preparations	Results	Findings	Citation
**Applied Voltage**	**(E-CE)C**	● (E-CE)C with M_n_ of 9.7 × 10^4^ g/mol, was prepared by a reaction of EC and Acrylonitrile with a DS of 2.1 for Ethyl and 0.37 for Cyanoethyl. ● THF was used as the solvent. ● Concentration of (E-CE)C/THF solutions is 17 wt%. ● Varying voltage between 0 and 100 kV. ● The diameter of orifice was 1.2 mm. ● TCD was 150 mm.	● Electrospinning did not start until the voltage was 20 kV. ● The crystallinity reached its maximum peak at 50 kV; it initially increased with increasing the voltage then it decreased. ● The average fiber diameter was as follow 5600, 6200, 9200, 10,700 nm according to 20, 30, 40, 50 (kV) respectively	● The crystallinity of the fibers was initially increased with increases in the voltage till it reach maximum value but then decreased with further increases in voltage. ● The average diameter of the fibers increased, and the diameter dispersion was broadened with voltage increases.	[27]
	**CA & EC**	● Polymer blends of CA + EC solutions with a concentration of 10% (2:1, w/v) with 2:1 (v/v) either Acetone-DMF, or Acetone-DMAC individually. ● Mechanical stirring and persistent heating (50 ± 1.8 °C) were applied for 12 h. ● Voltage was varying between 0 and 60 kV. ● 5 mL syringe with an inner diameter of 0.5 mm. ● The feed rate was at 0.5–1.0 mL/h. ● Under ambient conditions (21 ± 2 °C) and relative humidity (57 ± 3%). ● TCD was 150 mm.	● Applying a voltage of 9, 10, 11, 12 KV on: 1. Acetone + DMAc: It was noted that the conductivity increased with the increase of the applied voltage. 22, 24, 26, 28 S/cm respectively. The average fiber diameter was 717.4 ± 24, 730 ± 24, 757.7 ± 39, 1030.08 ± 42 nm respectively. 2. For Acetone + DMF: The conductivity and appearance of small beads increased by increasing the applied voltage. 20, 21, 22, 23 S/cm respectively. The average fiber diameter was 759.02 ± 29, 781.2 ± 38, 849.57 ± 23, 936.7 ± 28 nm respectively.	● By increasing voltage, the conductivity of the solution increase, that is due to the increase in the electrostatic forces on the jet. ● The fibers' diameter increases with the increase of the applied voltage.	[42]
	**Chitosan-collagen**	● Collagen I (mol wt, 0.8–1x10^5^ Da) and Chitosan (85%, deacetylated, Mɳ,ca. 10^6^) with (1:1) (w/w) in HFIP/TFA of (90/10, v/v). ● 8% (w/v) was used. ● a syringe of 5 mL with an orifice diameter of 0.46 mm ● Varying voltage: (12 kV–28 KV with 4 KV increment). ● Fixed TCD of 110 mm. ● Feed rate of (0.8 mL/h).	● The diameter of the resulting fiber doesn’t significantly change with the varying voltage. ● The average diameter was as follow 700, 750, 550, 700, 700 nm according to 12, 16, 20, 24, 28 kV.	● Voltage doesn’t have a significant effect on the fiber diameter.	[43]
	**Chitosan-Collagen & PEO**	● Low M_w_ chitosan was used with collagen of type I. ● 2.5 wt% of chitosan and 0.5 wt% collagen. ● A mixture of chitosan-collagen in glacial acetic acid of (99.7% purity) with 90% (v/v). ● PEO was added to the solution with 2.5 wt% concentration and with PEO:chitosan-collagen 10:90 (v/v). ● A 5 mL syringe was used. ● Varying voltage between 0 and 20 kV. ● Varying flow rate between 0.5 and 1.5 mL/h by increment of 0.5 mL/h. ● Varying TCD between 150 and 250 mm by 50 mm increment.	● No jet formed at voltage lower than 5 kV. ● When the voltage reach 7 kV Taylor cone starts to form. ● At 8–10 kV a stable Taylor cone was formed. ● For a voltage above 25 kV the voltage become unstable and splitting started. ● All the above values of voltage were tested with the different values of flow rate and TCD and gave the same results.	● There is a critical value for the applied voltage at which fibers start forming	[44]
	**CA**	● Dissolving an appropriate amount of CA (MW ∼100,000 Da, acetyl content∼39.7 wt%) in acetone by stirring at 20^O^C. ● CA Concentrations 15% (w/v). ● Using 10 mL syringe with a 22-gauge blunt needle. ● Feed rate was kept constant at 2 mL/h. ● Constant ambient temperature (20 °C). ● Applied voltage 8–16 kV. ● TCD 100 mm.	● For applied voltage of 8 KV the average fiber diameter was 749 nm. ● For applied voltage of 16 KV the Average fiber diameter was 823 nm.	● With increasing the applied voltage, the average fiber diameter increases.	[45]
**Flow Rate**	**Chitosan-collagen**	● Collagen I (mol wt, 0.8–1x10^5^ Da) and Chitosan (85%, deacetylated, Mɳ,ca. 10^6^) with (1:1) (w/w) in HFIP/TFA of (90/10, v/v). ● Constant solution concentration of 8% (w/v) was used. ● a syringe of 5 mL with an orifice diameter of 0.46 mm ● Varying feed rate. ● Fixed voltage of 16 kV. ● Fixed TCD of 110 mm.	● The average diameter of the fiber was 700, 700, 750, 800, 800 nm according to 0.36, 0.48, 0.6, 0.72, 0.84 mL/h	● Feed rate affects the size and the homogeneity of the fiber as it controls the volume of drawn solution from the needle. ● The average fiber diameter increased with the increase of the feed rate.	[43]
	**(E-CE)C**	● (E-CE)C with M_n_ of 9.7 × 10^4^ g/mol with a DS of 2.1 for Ethyl and 0.37 for Cyanoethyl. ● THF was used as the solvent. ● Concentration of (E-CE)C/THF solutions is 17 wt% ● The applied voltage is 30 kV ● The diameter of orifice was 1.2 mm. ● TCD was varying.	● At a TCD greater than 250 mm, fibers could not be collected. 200 mm is considered an ideal TCD for the experiment. ● The average diameter of the fiber was as follow 5100, 4400, 3800, 1900 nm according to 50, 100, 150, 200 mm	● The average diameter of the fibers decreases with the increase in the TCD. ● TCD had to be adjusted as it’s a limited parameter which at a specific value won’t allow any formation of fiber.	[27]
**Tip to Collector Distance**	**Chitosan-collagen**	● Collagen I (mol wt, 0.8–1x10^5^ Da) and Chitosan (85%, deacetylated, Mɳ,ca. 10^6^) with (1:1) (w/w) in HFIP/TFA of (90/10, v/v). ● Constant solution concentration of 8% (w/v) was used. ●a syringe of 5 mL with an orifice diameter of 0.46 mm. ● Constant feed rate (0.8 mL/h). ● Fixed voltage of 16 kV. ● Varying TCD from 80 mm to 160 mm	● In case of low ambient humidity, the increase in the TCD decrease the average diameter of the fiber and vice versa, the TCD also affects the fiber homogeneity as when the TCD is too small the fiber will be non-uniform. ● The average fiber diameter was 550, 500, 650, 650, 700 nm according to TCD as follow 80, 100, 120, 140, 160 mm	● The TCD doesn’t affect the size and the homogeneity of the fiber directly as it depends on many other factors like ambient humidity and the evaporation of the solution.	[43]
	**Chitosan & gelatin**	● Chitosan of (degree of deacetylation 0.85, M_W_ 110 kDa) 3% (w/v) and Gelatin 30% (w/v) in 80% Acetic acid. ● Volume Ratio of Chitosan/gelatin was 80:20. ● Solutions were stirred for 20 h. ● Diameter of the nozzle was 0.1 mm ● Voltage was 12 kV. ● Flow rate 0.1 mL/h ● TCD varied from 80 to 240 mm.	● For different TCD there was no difference in the alignment, number of beads, and fiber distribution. ● At 80 mm, the average fiber diameter was 200 ± 40 nm. ● At 160 mm, the average fiber diameter was 180 ± 20 nm. ● At 240 mm, the average fiber diameter was 160 ± 20 nm.	● The average fiber diameter decreases by increasing TCD.	[46]
**Orifice Diameter**	**(E-CE)C**	● (E-CE)C with M_n_ of 9.7 × 10^4^ g/mol with a DS of 2.1 for Ethyl and 0.37 for Cyanoethyl. ● THF was used as the solvent. ● Concentration of (E-CE)C/THF solutions was 17 wt%. ● The applied voltage is 30 kV. ● TCD was adjusted to be 150 mm. ● Varying orifice diameter.	● For Orifice Diameters of (0.7, 0.9 and 1.2) mm the average fiber diameter was (1000, 1100 and 2600) nm.	● The average fiber diameter increases by increasing the Orifice Diameter.	[27]

#### 4.2.2 Effect of flow rate

**Fig. 4:**
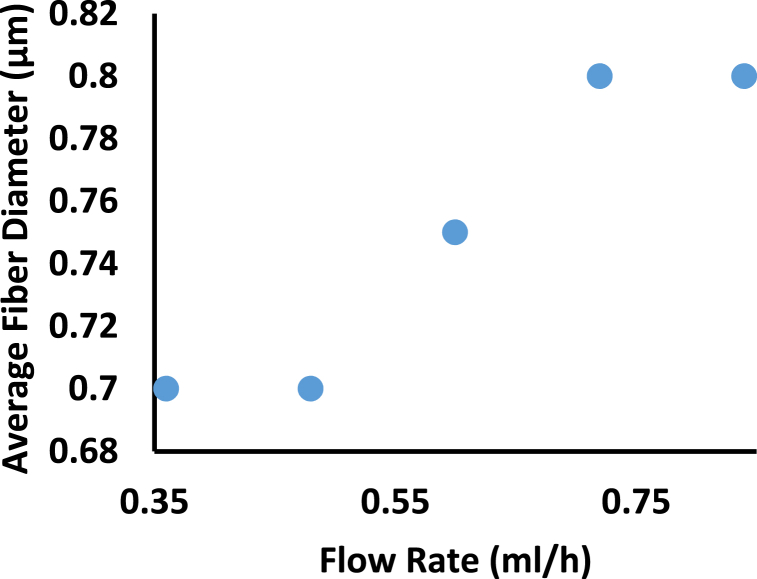
Effect of Flow Rate on Average Fiber Diameter for 8% of Chitosan Collagen dissolved in HFIP/TFA [43].

Increasing the flow rate above a critical threshold causes an increase in fiber diameter, as in Fig. 4 [43] and bead formation (Table 2) and this happens because the nanofiber jet is not completely dried during its journey between the needle tip and the metallic collector, as the pumped solution has no time to dry out and form intact fiber [43]. To maintain a balance, it is preferable to minimize the flow rate. This allows the formation of a stable jet cone and on sometimes a receded jet. Receded jet exits from the needle’s inside without forming a droplet or cone. These jets are not stable, and they are regularly replaced by cone jets during the electrospinning process [19,49].

#### 4.2.3 Effect of needle to collector distance

**Fig. 5:**
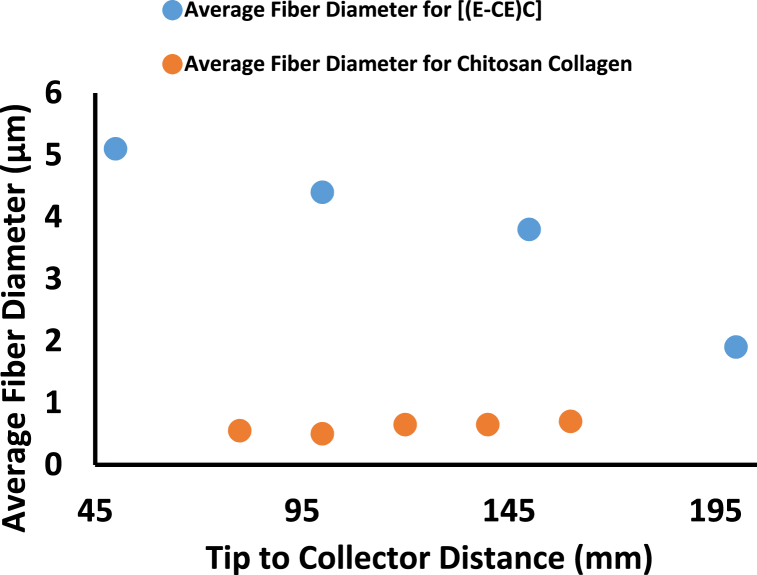
Effect of TCD on Average Fiber Diameter for 17% of [(E-CE)C]/THF Solution and 8% of Chitosan Collagen dissolved in HFIP/TFA [27].

The needle to collector distance affects deposition time, evaporation rate, and whipping or instability interval so, the nanofiber morphology depends on it. To create smooth and uniform electrospinning nanofibers, a critical distance must be maintained, as explained in Table 2, and any alterations on either side of the critical distance will influence the morphology of the nanofibers [19,25,49,50]. The average fiber diameter decreases by increasing TCD (see Fig. 5) [27]. Extreme reduction of collecting distance leads to higher jet stretching and elongation, resulting in lower fiber diameters [27,43]. The jet may not have enough time to dry if the collecting distance is too short, resulting in a non-uniform fiber sample. Suitable flight duration to allow the solvents to evaporate is a critical requirement for the polymer solution jet. The fiber can be obtained within the acceptable collecting distance. Collecting distance may affect some factors such as the evaporation of the solvent, electric field strength, and so forth, as explained in Table 2.

#### 4.2.4 Effect of orifice diameter

**Fig. 6:**
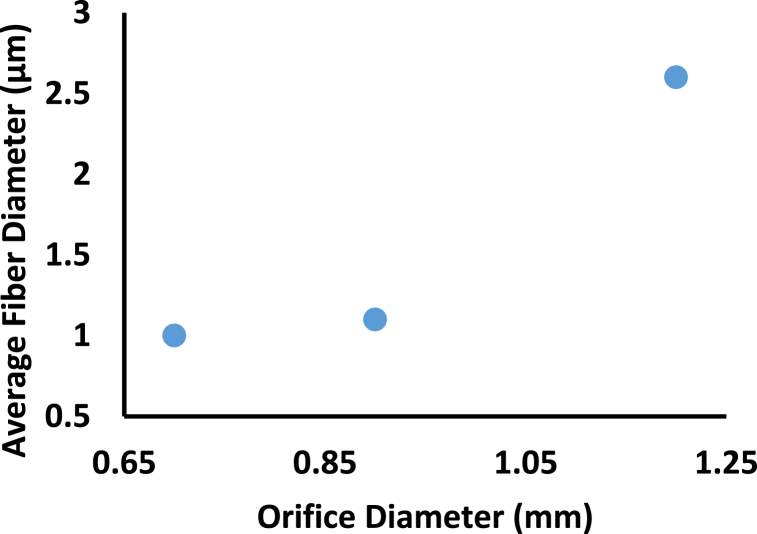
Effect of orifice diameter on average fiber diameter for 17% of [(E-CE)C]/THF solution [27].

The average fiber diameter increases due to the increase in the orifice diameter, see Fig. 6 [27]. As the radius of the droplet decreases, the surface tension of the droplet increases which, in turn, reduces both the initial acceleration and average velocity. So, it takes a long time for a jet to travel from the anode to the collector. The charged solution jet has a higher chance to split and elongate. Using a narrow aperture, tiny diameter fibers with a limited dispersion may be produced [27].

### 4.3 Ambient parameters (effect of humidity and temperature)

**Fig. 7:**
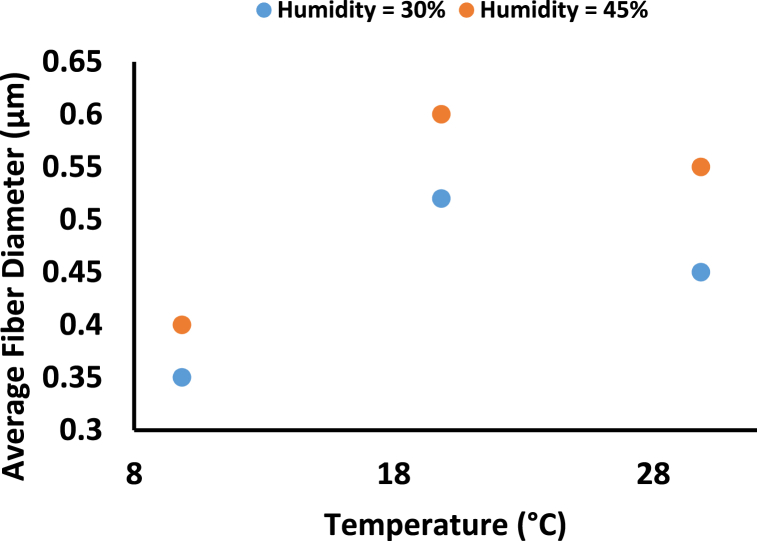
Effect of Humidity on Average Fiber Diameter for 17% Cellulose Acetate dissolved in (2:1 v/v) aceton:DMAC [47].

**Fig. 8:**
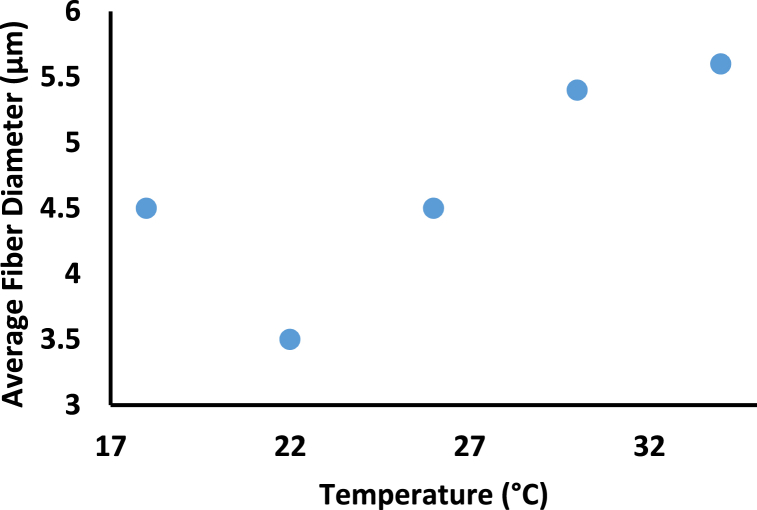
Effect of Temperature on Average Fiber Diameter for 17% [(E-CE)C]/THF solution [27].

Environmental (ambient) variables such as relative humidity and temperature, in addition to machine and material characteristics, have recently been shown to impact the diameter and shape of nanofibers. The average diameter increased by increasing humidity, see Fig. 7 [47]. Precipitation will result in the formation of non-woven fibers. Humidity leads to a variation in the diameter of the nanofibers caused by the varying solidification of the charged jet and, thus, leads to forming bead fibers for individual polymers and almost no electrospinning for the blends [19]. An Increase in the spinning temperature results in an increase in the fiber diameter (Fig. 8) [27] and wider diameter distribution. When the solution temperature becomes higher, the diameter of the fibers also increases, as explained in Table 3 and diameter distribution widens [27]. There is an optimal value of spinning temperature at 22 °C that has a minimum average fiber diameter (see Fig. 8) [27]. A low spinning temperature leads to a low solvent evaporation speed, and the solvent cannot be entirely volatilized when the charged solution jets reach the collector. Agglutination of solution jets on the collector can result in increased fiber diameter and a broader diameter dispersion. Because of the rapid evaporation of the surface solvents at higher temperatures, the charged solution jets have less time to divide and elongate throughout their flight. At the optimal spinning temperature, which was 22 °C in this case, the solution jets solidify by solvent volatilization when they arrive at the collector, and the jets have enough time to split throughout their flight to the collector.

**Table 3:** Effect of Ambient parameters (humidity and temperature) on the average fiber diameter.

Ambient parameters					
Parameter	Material	Methods and Preparations	Results	Findings	Citation
	**(E-CE)C**	● (E-CE)C with M_n_ of 9.7 × 10^4^ g/mol with a DS of 2.1 for Ethyl and 0.37 for Cyanoethyl. ● THF was used as the solvent. ● Concentration of (E-CE)C/THF solutions is 17 wt% ● The applied voltage was 30 kV ● The diameter of orifice was 1.2 mm. ● The TCD was adjusted to be 150 mm.	● The percentage of fibers with a diameter smaller than 4 μm is 77% at a temperature of 22 °C. ● The average fiber diameter was 4500, 3500, 4500, 5400, 5600 nm according to spinning temperature 18 °C, 22 °C, 26 °C, 30 °C, 34 °C	● The average fiber diameter increases with the increase of the spinning temperature.	[27]
**Humidity and Temperature**	**CA**	● acetone:DMAc with 99.5% purity for the DMAc. ● Cellulose acetate solutions with 17 wt% was dissolved in 2:1 v/v of acetone:DMAc. ● stirred at 25 c° for 4 h s. ● TCD was 120 mm. ● Flow rate of 1 mL/h. ● Fixed voltage of 15 kV.	● At high RH% rates above (60%), it is not possible to produce well-shaped nanofibers. ● Only at RH 45%–60% a complete nonwoven fiber was formed; at the rest of the other conditions, a wet spot was found in the middle. ● For 45% relative humidity the average fiber diameter was 400, 600, 550 nm for 9.85 °C, 19.85 °C, 29.85 °C. ● For 30% relative humidity the average fiber diameter was 350, 520, 450 nm for 9.85 °C, 19.85 °C, 29.85 °C respectively.	● The average fiber diameter increases with the increase of RH at the same temperature. ● According to the chemical nature of CA, an increase in humidity, water absorption and precipitation will increase the chance of formation of nonwoven fibers.	[47]
	**Chitosan**	● Chitosan of 80 mg/mL dissolved in TFA and DCM of relative concentration 70/30 (v/v). ● TCD was 120 mm. ● Voltage was 17 kV ● Flow rate was 0.2 mL/h (20,22, 27,32) °C (solution temperature).	● A change in the morphology from bead connected fibers to uniform fibers was observed. ● Facilitating the electrospinning process, as a result of decreasing the Chitosan viscosity.	● The uniformity of the fiber increases with the increase of the solution temperature. ● Increasing the solution temperature can cause a decrease in the Chitosan viscosity.	[48]

## 5 Conclusion

Electrospinning is a reliable and widely used method that has been implemented in various fields. Cellulose and chitosan nanofibers are particularly popular due to their numerous applications. Cellulose nanofibers, for instance, find use in tissue engineering and medical implants, while chitosan nanofibers are used for water purification and air filters. To improve the characteristics of electrospinning nanofibers, extensive research has been conducted to explore the impact of various parameters such as concentration, viscosity, type of solvent, surface tension, conductivity, applied voltage, flow rate, TCD, orifice diameter, humidity, and temperature. Based on the findings, it is concluded for:

Cellulose:

• The average fiber diameter increased gradually with the increase in solution concentration.
• With the increase of the applied voltage, the average fiber diameter increased.
• Due to the increase in TCD, the average fiber diameter decreased gradually.
• The average fiber diameter increased with the increase of the orifice diameter.
• With the increase in spinning and solution temperature, the average fiber diameter gradually increased.
• the average fiber diameter increased with the increase in humidity.

Chitosan:

• The average fiber diameter was almost constant with the increase in the applied voltage.
• The average diameter gradually increased with the increase of the flow rate.
• The average fiber diameter is nearly fixed by the increase of the TCD.

According to previous findings, it was discovered that to produce fine fibers from cellulose and chitosan, optimized monitoring of the different parameters (material, machine, and ambient parameters) should be performed, and this will be accomplished through the study of changing these parameters all at once rather than individually.

## Author contribution statement

All authors listed have significantly contributed to the development and the writing of this article.

## Funding statement

This research did not receive any specific grant from funding agencies in the public, commercial, or not-for-profit sectors.

## Data availability statement

Data will be made available on request.

## Declaration of competing interest

The authors declare that they have no known competing financial interests or personal relationships that could have appeared to influence the work reported in this paper

## Abbreviations

IL: Ionic liquid
[C_2_min][OAc]: 1-ethyl-3-methylimidazolium acetate
DMF: Dimethylformamide
DMAc: Dimethylacetamide
TCD: Tip to collector distance
(E-CE)C: Ethyl– cyanoethyl cellulose
M_n_: Number average molecular weight
THF: Tetrahydrofuran
CMC: Carboxy-methyl cellulose sodium salt
PEO: Polyethylene oxide
M_W_: Molecular weight
DS: Degree of substitution
M_v_: viscosity average molecular weight
PVA: Poly(vinyl alcohol)
FA: Formic acid
DWD: Distilled water
DCMD: Dichloromethane
CA: Cellulose Acetate
EC: Ethyl cellulose
H-chitosan: Hexanoyl chitosan
HFIP: 1,1,1,3,3,3- hexafluoroisopropanol
RH: Relative humidity
BMIMAc: 1-butyl-3-methyl-imidazolium acetate
DMAA: N,N-dimethylacetamide
PEG: Polyethylene glycol
CB: Carbon Black
TFA: Trifluoroacetic acid
PLA: Polylactic acid
CMC: Carboxyl functionalized graphene oxide
GO: Graphene oxide
